# Continuation of the Evidence Delivered before the Committee of the House of Commons, in Support of Dr. Jenner's Application for Parliamentary Reward

**Published:** 1802-07

**Authors:** 


### *Continuation of the Evidence delivered before the Committee of the House of Commons, in Support of Dr. Jenner's Application for Parliamentary Reward.*

Continued from Vol. VII. pp. 489—496.

Mr. Cline was confulted upon the cafe of a child of Mr. Auftin at Clapton, with whom it was faid the cow-pox inoculation had failed; but from particular enquiries of the parents and nurfe, he was perfectly convinced the child had never received the vaccine difeafe; and this evidence Mr. Taylor, the Surgeon, who inoculated it, confirmed. He thinks that experience has fufficiently demonfteratd that perfons inoculated with the cow-pox, are incapable of receiving the fmall-pox; and he believes that in the inftances where the fmall-pox has been caught, and the patient has, before the coming out of the difeafe, been inoculated with the cow-pox, it mitigates the virulence of the fmall-pox. The vaccine difeafe is not contagious, nor does it create any blemifh on the human frame; nor does it excite fcrophula, or any other difeafe, which is fometimes the cafe with the inoculated fmall-pox.

In November, 1800, he performed the operation for the ftone on William Rench, a child in Ifaac's Ward of St. Thomas's Hofpital. In a few days after, hearing that this boy was in great danger of catching the fmall-pox, he directed that he fhould be inoculated with cow-pock matter, which took effect, and proceeded in the ufual manner: but in thirteen days after this inoculation, a few eruptions appeared that feemed to be variolous.

Admitting thefe eruptions were the true fmall-pox, the time of their appearance fhows the infection had been received before the child was inoculated with cow-pox matter; for the natural fmall-pox frequently does not appear until fixteen or eighteen days after the patient has been expofed to infection.

A fecond cafe was in November 1801. The child of Mary Solloway, in Mary's Ward of the fame Hofpital: this child was known to have been expofed to the infection of the fmall-pox, and therefore the mother permitted it to be inoculated with cow-pock matter; but in four days after, the fmall-pox appeared, and the difeafe was very fevere; however, the child recovered.

A third cafe was a patient of Dr. Lifter's, whofe mother had the fmall-pox. In fix days after the complaint had appeared in the mother, the child was inoculated with cow-pock matter, and the complaint from this inoculation proceeded as ufual; but in in about fifteen days a few eruptions appeared that were of a doubtful nature.

From the moſt minute inquiry, theſe are all the caſes which have occurred in St. Thomas's Hoſpital, where variolous eruptions have ſucceeded the vaccine inoculation, and in each of which there can be no doubt that the patients were expoſed to the infection of ſmall-pox previous to their being inoculated. (No. 33.)

Mr. DAVID TAYLOR, Surgeon, of Wooton-under-Edge, Glouceſterſhire, ſpoke to two caſes which had been brought before the Committee, as diſproving the efficacy of cow-pox in preventing ſmall-pox; the one of a child of Mr. Auſtin of Clapton, the other of a woman at Old Sodbury. With regard to the firſt, he had inoculated the child with vaccine matter himſelf, but did not ſee the progreſs of the diſorder, nor was the child attended by any medical perſon; but from the account given by thoſe who were with the child, he was apprehenſive at the time that the vaccine diſeaſe had not taken effect, and ſtrongly recommended that ſhe ſhould be inoculated for ſmall pox, which ſhe afterwards caught in the natural way. He ſtated, that a full and minute inveſtigation of the ſecond caſe had been made by five or ſix perſons, who were unanimous in their opinion that the woman at Old Sodbury had never had the cow-pox. His own practice in vaccine inoculation has been conſiderable, and he has inoculated about 2000 perſons without a ſingle failure; nor has he met with any ulcerations, humours, or diſeaſes following it, ſuppoſed to be excited by it. He has inoculated a large proportion of his patients with variolous matter afterwards, without any diſeaſe being produced. He further ſtated, that he was acquainted with the extent of Dr. Jenner's medical practice before he left Glouceſterſhire, where he was ſituated in a very populous neighbourhood, without any practiſing phyſician within 16 miles; well ſupported, and of courſe in the moſt conſiderable practice; and he thought that in conſequence of his quitting his ſituation in the country, and coming to town, he had leſſened his income moſt conſiderably, as two phyſicians had ſucceeded to the ſituation which Dr. Jenner had left, both of whom are in conſiderable practice, and of courſe Dr. Jenner's former ſituation cannot be re-attainable. (No. 40.)

Mr. JORDAN, Member of the Royal College of Surgeons, two or three years ago inoculated between one and two hundred with vaccine matter; ſome matter was received by him from the Apothecary of the Inoculation Hoſpital (over which Dr. Woodville preſides) for vaccine, which proved to be to be variolous; the patients, not being prepared for it, were very ill, but recovered; he has avoided thefe miftakes fince, by taking the matter himfelf from the patient; and has learnt by Dr. Jenner's publication how to diftinguifh and felect the proper time for taking it, fince which no miftake of the kind above mentioned has occurred. He is of opinion that errors of that kind brought vaccine inoculation for a time into difrepute. (No. 25.)

Mr. GARDNER has known Dr. Jenner more than twenty-two years, and been in the conftant practice of hearing his medical opinions and difcoveries. It was in the month of May, 1780, that Dr. Jenner firft informed him of the particular nature of the cow-pox as a fure preventive from fmall-pox, and the theory he had framed on the fubject; declaring his full and perfect confidence that it might be continued in perpetuity of inoculation from one human being to another, in the fame way with the fmall-pox, and in time fuperfede that difeafe. (No. 26.)

Dr. THORNTON, Phyfician to the Mary-le-Bone Difpenfary, ftated the cafe of two children belonging to Lord Somerville's coachman, whom he inoculated three years ago, with what he fuppofed to be true cow-pox matter; the matter from which the inoculation was performed at that early period of vaccine inoculation, was taken indifcriminately as long as there appeared a puftule from whence matter could be procured, he being unacquainted at that time, that the cow-pox inoculation ceafed to produce the difeafe after a certain period, which was known to Dr. Jenner, and publifhed by him, and forms one of the important difcoveries refpecting the new practice; he was fome time afterwards informed that thefe two children had the fmall-pox, and upon examining their arms, there were found no fcars, which is a criterion that thefe children had not had the true cow-pox, and he was confirmed in this belief by the mother of the children declaring that the puftules had advanced more rapidly than in the true cow-pox; this cafe appears to him important, as exhibiting a proof that all other cafes adduced againft the general principle of fecurity from vaccine inoculation, muft arife from want of acquaintance of the inoculator with the period when to take the matter; which difficulty he deems to be now completely done away, by Dr. Jenner having elucidated a fubject before involved in much obfcurity. He further ftates, that matter taken from a puftule, which was a week old, never failed to produce the true cow-pox; but in the aforementioned inftance of the two children, he has great reafon to believe it was taken the fourteenth day, or later: he ftates another fource of fpurious cafes in the lancet being corroded with the cow-pox matter, on which it is placed; he inoculated a week before fome patients from the fame matter with which he inoculated the afore-mentioned children, who went through the difeafe in a regular way; one patient in particular has been, during thefe laft three years, inoculated with fmall-pox matter at leaft twelve different times; he has even flept with a perfon who died of the natural fmall-pox, and has been otherways expofed, but could not take the infection; he fays, when he was in the north, at Lord Lonfdale's, he inoculated upwards of one thoufand perfons, and completely fatisfied himfelf, and all the medical practitioners in that part of England, that the cow-pox was a mild difeafe, hardly deferving that appellation; not contagious, never disfiguring the perfon, never producing blindnefs, never fatal, nor exciting other difeafes; equally fafe, whether during the period of pregnancy, or the earlieft infancy, or extreme old age. (No. 20.)

Earl of BERKELEY ftated, That his youngeft fon was inoculated with the cow-pox by Dr. Jenner at fix months old, and went regularly through its courfe; about a year after a maid fervant in the family caught the fmall-pox in the natural way, and was attended by Mr. Robert Pope, furgeon, from Staines, who pronounced the girl to be in a very dangerous fituation; having in the houfe at that time three perfons who had been inoculated with the cow-pox, the child above-mentioned, a maid fervant, and a little girl, and being defirous of proving the efficacy of vaccine inoculation, he fent for Dr. Jenner, and permitted him to inoculate the child and one of the girls with variolous matter, taken from the maid fervant; the fmall-pox took no manner of effect on either of them (the girl had been inoculated with vaccine matter four years before) the other girl that was not inoculated attended on the maid fervant the whole time until her death, and refifted the infection.—The effluvia in that part of the houfe was fo offenfive, that all the fervants were obliged to be removed to another part of the houfe. Lord Berkeley further ftated, That there is an old fervant now in his family, feventy-two years of age, who had the cow-pox, from milking cows, when a boy of fifteen, who has never been in the leaft cautious in guarding againft the fmall-pox, but has expofed himfelf repeatedly, without being fenfible of its effects; and Lord Berkely once faw him, himfelf, fitting next a boy who had the fmall-pox vifibly out upon him. (No. 6.)

ROBERT POPE, furgeon, at Staines, attended the fervant maid from whom Earl Berkeley's fon and the girl were inoculated with variolous matter; he depofed to the virulence of the difeafe, and to her death in confequence. His opinion was was not favourable to vaccine inoculation on the firſt publication of it; but he is ſince pretty fully convinced, that if properly conducted, it is a preventive of ſmall-pox, and he has practiſed it himſelf with ſucceſs. (No. 7.)

The Rev. G. C. JENNER is converſant in the practice of vaccine inoculation, and has inoculated three thouſand perſons, without meeting with one unfavourable caſe, although he has inoculated perſons from the earlieſt infancy to eighty years of age, and under thoſe circumſtances in which it would not be prudent or indeed ſafe to inoculate with variolous virus, ſuch as children at the time of dentition, and women in every ſtage of pregnancy, from the firſt month to the laſt week. Upwards of two hundred of his patients have ſince been inoculated with active ſmall-pox matter, and at leaſt an equal number expoſed to contagious effluvia, but in no one inſtance was the ſmall-pox produced. On the arms of ſome thoſe inoculated with ſmall-pox, a ſlight local inflammation ſhewed itſelf, which diſappeared in the courſe of four or five days; ſome of theſe perſons were put to the teſt of the ſmall-pox, after a period of a year. The perfect puſtule is always to be diſtinguiſhed from the imperfect or ſpurious, by thoſe who have paid a proper attention to the practice of vaccine inoculation. He believes that vaccine inoculation will frequently ſuperſede the infection of the ſmall-pox, when the patient has been expoſed to a variolated atmoſphere previous to the inoculation, in confirmation of which he related a caſe under his own immediate obſervation. A boy, infected with the natural ſmall-pox, came home to his father's cottage; four days after the eruption had appeared upon this boy, the family (none of whom had ever had the ſmall-pox) conſiſting of the father, mother, and five children, were inoculated with vaccine virus; on the arm of the mother it failed to produce the leaſt effect, and ſhe had the ſmall-pox; but the ſix others had the cow-pox in the uſual mild way, and were not affected with the ſmall-pox, although they were in the ſame room, and the children ſlept in the ſame bed with their brother, who was confined to it with the natural ſmall-pox, and ſubſequently they ſlept with their mother. He is of opinion, that if the practice of vaccine inoculation is univerſally adopted, it will in a ſhort time annihilate the ſmall-pox. He has known many inſtances of the infection not taking in the early part of his practice, owing to his uſing vaccine virus taken at too advanced a ſtage of the diſeaſe, but ſince he has made it a rule never to inoculate with matter after the eighth or ninth day of the diſeaſe, he has ſeldom met with a failure; he inoculated two hundred and thirty-eight individuals on the ſame day, with recent fluid virus, taken on the eighth day of the difeafe, and every one of them had the cow-pox in the moft perfect manner. The progrefs of the cow-pox is in general uniform; he has feen a few exceptions, one or two cafes have occurred when the progrefs of the difeafe has been retarded for at leaft a fortnight before there were any vifible appearances of the inoculation having fucceeded; the courfe of the fpurious diforder is univerfally quicker than the perfect, fo as to form a certain criterion between the forts in every cafe which has come under his obfervation; when the puftule affumes the genuine character, the patient may be confidered as fafe from any future attack of fmall-pox, although there has been no apparent conftitutional indifpofition. (No. 8.)

Dr. JOSEPH MARSHALL, Phyfician Extraordinary to the King of Naples, firft began to inoculate in the fummer of 1799, in Gloucefterfhire, having received inftructions on this fubject from Dr. Jenner. In July, 1800, recommended by Dr. John Walker, who affifted him in fome of thefe inoculations, he commenced this practice on board his Majefty's fhip the Endymion, eleven of whofe crew were inoculated, and went through the vaccine difeafe without any remiffion of their ordinary duty, or any deprivation of their ufual allowance of wine or provifions; he alfo inoculated fuch foldiers of the garrifon of Gibraltar, as had not had the fmall-pox; the plague, at this time, prevented the garrifon from receiving their ufual fupplies of frefh provifions from Barbary; and Spain was fhut againft them by the war; their food in confequence was principally falt provifions fent them from England, and they generally indulged in drinking new wine; this diet, added to the exceffes which foldiers ufually commit, put the cow-pox to a fevere trial, efpecially when it is further confidered that they, whilft under inoculation, performed their ordinary regimental duties; and fo far was the cow-pox from preventing their doing this, that not a fingle cafe occurred where any application was requifite to the inoculated part, though the heat of the atmofphere was frequently upwards of ninety degrees; in corroboration of which the Surgeon Major's certificate was produced. At Minorca the fame fuccefs attended the inoculation, where it was alfo generally introduced amongft the inhabitants; and their medical men were inftructed in the practice; fuch feamen alfo on board of the Britifh fleet, under the command of Admiral Lord Keith, as had not had the fmall-pox, were inoculated with the cow-pox. At Malta, its practice was alfo generally introduced both among the troops and inhabitants; and an hofpital, called the Jennerian Inftitution, was eftablifhed by the governor, for the inoculation inoculation of the poor; in this iſland the ravages of the ſmall-pox had always been dreadful, and ſome of the men of war, then in the harbour, had the ſmall-pox on board, and had buried ſeveral men; this apprehenſion was alſo entertained by the Admiral and General Sir Ralph Abercrombie, who each iſſued general orders for the inoculation of ſuch ſeamen and ſoldiers under their reſpective commands, as had not had the ſmall-pox. A certificate, confirming the above facts, was delivered in, ſigned by Sir Alexander Ball, governor of Malta. In Sicily, the ſmall-pox had been, if poſſible, ſtill more fatal than in Malta, for the computation of deaths, occaſioned by it in the year preceding his arrival, exceeded eight thouſand in the city of Palermo alone; the introduction of the cow-pox was therefore received with enthuſiaſm, and an hoſpital, ſimilar to that at Malta, was immediately eſtabliſhed by his Sicilian Majeſty; and although the ſmall-pox, ſoon after his arrival in the city, again appeared, it was immediately ſtopped by the practice of vaccine inoculation, which was alſo extended through the whole iſland; the benefits received at Palermo from the cow-pox excited a great wiſh for its practice in Naples, where the ſmall-pox has always been conſidered as very fatal; an hoſpital was alſo there eſtabliſhed by his Majeſty, and the practice of vaccine inoculation was ſpeedily adopted throughout the whole kingdom. His Majeſty having commanded that children to be inoculated, attended by ſurgeons to be inſtructed in the practice, ſhould be ſent from each province to the hoſpital at Naples, to carry both the knowledge of the diſeaſe, and the practice of it into their reſpective provinces; on his leaving Naples, the witneſs received very honourable teſtimonials from his Sicilian Majeſty, which were produced. He alſo extended this practice to other parts of Europe, to Rome, Leghorn, and Genoa, and in every inſtance, where tried, he found it reſiſt the infection of ſmall-pox. He never heard that any ſuch mode of inoculation had been practiſed or known in thoſe countries before; and as an example of the diſbelief entertained by the medical men of Naples, he related a trial which they inſtituted ſoon after his arrival there, and without his knowledge, at the Foundling Hoſpital; where they firſt inoculated with the cow-pox, a conſiderable number of children; and after they had paſſed through the diſeaſe, expoſed them to all poſſible modes of infection of the ſmall-pox, both by inoculation, and by making them ſleep in the bed with people infected with the ſmall-pox. This trial, which had excited the attention of the whole city, completely eſtabliſhed the reputation of the cow-pox; and they appointed a deputation to him, publicly to expreſs their conviction of its efficacy. He believes the number inoculated under his direction was upwards of ten thoufand, many of whom were fubjected to every poffible means of variolous infection, which all of them refifted. He never did obferve the vaccine inoculation to introduce or excite any other difeafe; on the contrary, children in a weak ftate of health have immediately, after paffing through the vaccine inoculation, begun to thrive and became vigorous. He is of opinion, that the fpurious or imperfect fort is eafily diftinguifhable from the perfect difeafe, and that a perfon who has once feen the true cow-pox puftule can never be miftaken. (No. 9.)

Mr. JOHN GRIFFITHS, Surgeon to the Queen's Houfehold and to St. George's Hofpital, has inoculated upwards of fifteen hundred perfons with vaccine matter, not one of whom has had any untoward fymptom; among them three of his own children, at various periods, within three years. (No. 10.)

Dr. SKEY, Phyfician to the Worcefter Infirmary, ftated, That in the fpring of laft year the fmall-pox being generally and fatally epidemic in the city of Worcefter, he feized the opportunity of inoculating a number of children with the cow-pox; that in the diftrict where he inoculated the greateft number, the fmall-pox contagion ceafed to exert its influence, and the number of victims gradually diminifhed; that in every cafe which he witneffed, the inoculated cow-pox was incomparably lefs fevere than the natural fmall-pox; that none of thofe patients whom he inoculated with the vaccine matter, received the fmall-pox afterwards, although they were conftantly expofed to the fmall-pox contagion, and although cafes of the two difeafes not unfrequently occurred under the fame roof, and at the fame time; that he had never yet known a cafe in which any confiderable degree of hazard was incurred by the vaccine difeafe, and that he had not met with a fingle inftance in which, after a fecond or third inoculation, he did not fucceed in producing the vaccine difeafe. (No. 14.)

Dr. CROFT has paid particular attention to vaccine inoculation ever fince its firft introduction; from that time his own children have been inoculated with it, and he has uniformly recommended it to his patients; he has even recommended infants to be inoculated at the end of the month, but he never dared to recommend the inoculation of the fmall pox earlier than at two years of age, except under very particular circumftances. Upon being defired to relate what he knew concerning the inoculation of a child of Sir George Dallas, he ftated, that he recollected a child of Sir George Dallas's being inoculated with the vaccine difeafe by Dr. Jenner; he believed, in five five days from the time it was inoculated, it broke out with the fmall-pox; it went through the difeafe rather favourably; he was not called in to the child till about the third or fourth day of the eruption of the fmall-pox, when the arm inoculated appeared in the ftate one fhould naturally expect to find it from the fifth to the feventh day. He imagined that the inoculation of this child with the vaccine matter might have fome effect in abating the violence of the natural fmall-pox, the eruption not being equal to what might have been expected from the violence of the firft attack of fmall-pox fever. Sir George Dallas has fince had an infant of one month old inoculated with the vaccine difeafe. He had feen children, whofe arms had been confiderably inflamed from being inoculated with matter taken from under the vaccine fcab as late as the fourteenth day, but does not know why this fhould be called a fpurious fort of cow-pox, as they had none of the characters of vaccine difeafe. He is of opinion, that if the vaccine inoculation were generally introduced, it would be productive of greater bleffings on mankind than any other difcovery that was ever made in medicine, as it would ultimately caufe the fmall-pox only to be remembered by name. (No. 15.)

Mr. JAMES SIMPSON, Surgeon to the Surry Difpenfary, and to the Magdalen Hofpital, has practifed vaccine inoculation, and has inoculated between fifty and fixty patients, and in no one cafe had any fymptoms occurred injurious to the part inoculated, or conftitution of the patients; and he believes them to be completely fecure from the fmall-pox. In one particular inftance where the patient, a child of nine months, was covered with a cruft commonly called the crufta lactea, which generally covers the body from head to foot, and had refifted the ufual remedies for that difeafe, but on the tenth day after the infection it began to difappear, and on the twelfth was wholly gone, during which time not a particle of medicine was given to it, and it continued in perfect health ever fince. (No. 16.)

---

Many other perfons, of the firft refpectability, gave evidence to the fame effect as thefe we have felected; and Dr. Jenner affured the Committee, that he was prepared to adduce any number they might choofe to examine. They informed him, that he had eftablifhed the allegations contained in his petition to their entire fatisfaction. And it does not appear that any member of the houfe, when the Report was taken into confideration, entertained the fmalleft doubt of the Petitioner's claim on the munificence of his country. We have never witneffed a more more unanimous and general difappointment than that which has been expreffed, not only by the profeffion but by the public at large, at the fmallnefs of the remuneration. Although the Minifter franks the Petition through the Houfe of Commons, he cannot frank the £.10,000 through the Exchequer, where we underftand that double tythes of the fum generally difappear. Some of our Readers may, perhaps, fuppofe that the contravening evidence, which tended to diminifh the Petitioner's claims, was of confiderable weight; or that the number of adverfe cafes or failures bore a large proportion to the fuccefsful ones. We can, however, affure them, that in our judgment, and we attended the examinations very diligently, there was not a fingle failure eftablifhed, though much induftry was employed in fearching for them; and the patience of the Committee in inveftigating every rumour, even though prefented in anonymous letters, has feldom been equalled. As the public, however, will doubtlefs be curious to know what could be alleged againft Dr. Jenner's claims, and as the Committee have publifhed the *whole* of the contravening evidence, though that in favour of the Petitioner was neceffarily abridged, on account of the number of repetitions it contained, we fhall give an abftract of it in our next.

